# To be or not to be: the importance of attendance in integrated physiology teaching using non-traditional approaches

**DOI:** 10.1186/1756-0500-4-360

**Published:** 2011-09-14

**Authors:** Beatriz Gal, Ignacio Busturia, Concepción Garrido

**Affiliations:** 1 Departamento de Ciencias Biomédicas Básicas, Universidad Europea de Madrid, Villaviciosa de Odón, Madrid 28670, Spain; 2 Departamento de Estadística e Investigación Operativa. Universidad Complutense de Madrid. Madrid 28034, Spain; 3 Departamento de Ciencias Morfológicas y Biomedicina. Universidad Europea de Madrid, Villaviciosa de Odón, Madrid 28670, Spain

## Abstract

**Background:**

There is increasing use of non-traditional methods like problem-based learning, team-working and several other active-learning techniques in Physiology teaching. While several studies have investigated the impact of class attendance on the academic performance in traditional teaching, there is limited information regarding whether the new modalities are especially sensible to this factor.

**Methods:**

Here, we performed a comparative study between a control group receiving information through traditional methods and an experimental group submitted to new methodologies in Physiology teaching.

**Results:**

We found that while mean examination scores were similar in the control and the experimental groups, a different picture emerge when data are organized according to four categorical attendance levels. In the experimental group, scores were not different between the 1st and the 2nd exams (P = 0.429) nor between the 2nd and the 3^rd ^exams (P = 0.225) for students that never or poorly attend classes, in contrast to the control group (P < 0.001). A score difference between attending students versus the absentees was maximal in the experimental versus the control group all along the different exams and in the final score.

**Conclusion:**

We suggest that class attendance is critical for learning using non-traditional methods.

## Background

Physiology teaching constitutes an essential part of medical and other health science training programmes. This matter is currently experiencing important reforms in most European countries regarding its curriculum design in order to meet the Bologna Declaration [1,2]. The Bologna process aims to create an European Higher Education Area by 2010 in order to make studies compatible and comparable amongst countries and to increase competitiveness and quality. Such calls for reforms are mirrored worldwide towards active learning approaches aimed to more actively involve students in the learning process [3].

Traditionally, physiology has been taught through classical lectures and laboratory practices with little focus on strengthening specific competencies and interdisciplinary interaction. For physiology teaching, these reforms are focused in adopting an integrated curriculum drawn along the lines of student-centered approaches aimed to assimilate the different physiological systems into a unified view [4-7]. This involves using non-traditional methods, including problem-based learning, team-working and several other active learning techniques [7-9], and new evaluation systems that successfully measure all the aspects of knowledge [10-13]. Such an approach seems essential nowadays to equip students with the competences required for their social and professional integration in the modern knowledge-based society.

Several studies have investigated the impact of class attendance on the academic performance [14,15]. Most of these studies were focused in traditional teaching methods and have reported that lecture attendance levels have an impact that ranges from moderate [16,17] to significant on academic performance [18]. However, there is a fundamental difference in knowledge delivery between traditional and non-traditional teaching, that could make academic performance more sensible to class attendance when active and student-based methodologies are in use.

Here we aimed to look at the impact of attendance on integrated non-traditional physiology teaching. We report the results of a comparative study between a control group receiving information through traditional methods and an experimental group submitted to new methodologies that meet the new European standards. Our results suggest a fundamental effect of attendance on academic performance when new methodologies are used.

## Methods

This study was approved by the ethics committee of the Universidad Europea de Madrid in compliance with the Helsinki Declaration. Academic scores were recorded blindly and personal data were not included, except for gender information. Students were informed on the purpose of the study at the beginning of the academic course and their consent was waived.

### Groups

Control group included students from two consecutive academic courses (2003/04 and 2004/05) in the Podiatric School at the Universidad Europea de Madrid. There was no difference of examination scores between the two academic years (p = 0.761, ANOVA) and data were pooled (42 students). The experimental group included students from the next two consecutive academic years (2005/06 and 2006/2007) from the same school, and it shared professor staff with control group. Again, there was no difference on exams scores (p = 0.510, ANOVA) and data from the two academic years were pooled (19 students). The control and experimental groups were age and gender balanced without statistical differences. Groups were also balanced in terms of innate ability or intelligence with similar pre-school scores. Baseline scores, in a scale from 0 to 10, were similar from both groups: 5.71 ± 0.48 versus 5.89 ± 0.71 for the control and the experimental group respectively, t-test p = 0.552). Therefore, both groups were homogenous in terms of age, gender, professor staff and baseline scores.

### Course organization

The control group received classical physiology lectures and laboratory practices. Physiology in the experimental group was taught through an integrated curriculum including problem-based learning (PBL), clinical case discussion, team working tasks, laboratory practices and classical lectures, being these last two activities similar to the control group. Most lecture themes were shared by the control and experimental groups, with the only exception of some introductory items and overlapping anatomy and physiology contents in the control group. Most of these items were further developed by students in the experimental group as part of team working tasks and home work. Laboratory practices were essentially similar between groups with the few exceptions of the remaining items that were transferred from classical lectures in the experimental group. These contents were developed by students in the experimental group as preparatory material. Using this approach we redistributed classical lecture credits making room for complementary PBL, clinical case discussion and team working tasks in the experimental group. The number of contact-hours (72 in total) was similar for both groups. Therefore, both groups received essentially the same contents although being distributed in different ways with students in the experimental groups having more active learning activities. For both groups, statements on the course organization, learning objectives, bibliography, text book material, activities and examination schedule were provided at the beginning of each academic year and they were used all over the course for guiding purpose. This guarantees that students from both groups have access to appropriate materials for self-directed study.

### Examination

Knowledge was measured with multiple choice tests in both groups. This represented 100% examination score for the control group and 60% for the experimental group. Examination questions were designed to test for learning objectives as clearly stated in the material given to the students, i.e. they were not necessarily linked with the lecture contents. Thus class attendance has similar impact in terms of examination design for both groups. Laboratory practices and PBL, clinical cases and team-working accounted for the remaining 40% in the experimental group. This allowed us having similar standards for contrast between groups by comparing multiple choice test outcomes. These activities were continuously evaluated throughout the year. Due to academic requirements there were four and three examinations for the control and experimental groups, respectively. Exam scores ran in a scale from 0 to 10. The final course score represented a mean of the partial examinations for both groups.

### Attendance scale

Attendance was directly recorded by the professor during all the activities. The Universidad Europea de Madrid applies no penalties for absence. We used four categories for looking at the impact of attendance in learning. To this purpose we computed the mean and the standard deviation (SD) of attendance per academic year in order to define the following four categories: never (students that never attend), poor (students attending less than the mean - 2SD), mean (mean ± SD) and frequent (mean + 2SD). We found no significant evolution on the attendance over the course of the year in neither group.

### Statistical analysis

Data were analyzed using parametric (ANOVA and Student-t test) and non-parametric (Mann-Whitney for two independent samples, and Friedman for two-way repeated measures). Normality was checked using the Kolmogorov-Smirnov Test.

## Results

We first compared the examination scores between the control and the experimental group. We found no difference between the final examination scores from both groups (Table 1, ANOVA, p = 0.510), neither for the partial exam scores with the only exception of the 2nd partial exam score which was different between groups at p = 0.034. However, a different picture emerged when class attendance was considered. We looked at the effect of class attendance by reorganizing data according to four categories: never, poor, mean and frequent (see Methods). There was no difference between attendance levels in the control versus the experimental group (Mann-Whitney, p = 0.685)

**Table 1:** Examination results in the control and experimental groups

	1st exam	2nd exam	3rd exam	4th exam	Final score
**Control: test**	4.85 ± 1.8	5.88 ± 1.9	4.72 ± 1.7	5.55 ± 1.8	5.20 ± 1.9
**Experimental: test**	4.25 ± 1.7	3.91 ± 1.6	4.12 ± 2.1	--	4.10 ± 1.8

All data as mean ± SD

*comparison between exams

Tables 2 and 3 summarize examination results in the control and experimental group according to this scale. In both groups exam scores significantly increased from the 1st exam for students that never attend to the last exams for attending students (P < 0.001; Tables 1 and 2), suggesting that class attendance has a similar beneficial effect over the scores for both groups. However, we found a largest impact of attendance on performance in the experimental versus the control group when comparing the score evolution of attending students versus their peers. In the experimental group, scores were not different between the 1st and the 2nd exams (p = 0.429) nor between the 2nd and the 3rd exams (p = 0.225) for students that poorly attend classes, in contrast to the control group where there was evolution (p < 0.001). This suggests that absentees poorly improve performance along the academic course in the experimental group. Indeed, the score difference between attending students versus their peers was maximal in the experimental compared with the control group all along the different exams and in the final score. This suggests that class attendance is critical for learning using non-traditional methods.

**Table 2:** Mean examination results according to attendance in the control group

attendance	1st exam	2nd exam	3rd exam	4th exam	Final score
never*	3.30	4.70	4.70	4.25	4.40
**poor**	3.87 ± 2.44	5.53 ± 3.32	4.27 ± 1.41	5.25 ± 2.45	4.30 ± 2.39
**mean**	4.24 ± 1.53	5.39 ± 1.43	4.15 ± 1.53	5.09 ± 1.52	4.47 ± 1.76
**frequent**	5.74 ± 1.64	6.52 ± 1.98	5.36 ± 1.91	6.16 ± 2.11	5.97 ± 1.68

All data as mean ± SD

*only one student never attended

**Table 3:** Mean examination results according to attendance in the experimental group

attendance	1st exam	2nd exam	3rd exam	Final score
**never**	--	--	--	--
**poor**	3.80 ± 2.51	4.91 ± 0.44	5.27 ± 0.87	4.66 ± 1.02
**mean**	3.90 ± 1.65	4.83 ± 1.18	5.03 ± 1.42	4.55 ± 0.79
**frequent**	6.42 ± 1.23	6.65 ± 1.31	6.64 ± 1.53	6.56 ± 1.27

All data as mean ± SD

## Discussion

We have compared the impact of class attendance on academic performance depending on the use of traditional (control group) or non-traditional methods (experimental group) in Physiology teaching. We found that in the experimental group there was significant difference in exam scores between attending students and those who poorly attend classes. Moreover, a statistically significant increase of performance across different exams is present in the control but not in the experimental group for absentees. This supports the idea that students that poorly attend classes hardly compensate their performance when non-traditional methods are used and points towards the importance of class attendance in such a teaching modality. One may argue that there are similar beneficial effects of attendance in both groups since poor attendees improve more than attending students in the control and experimental groups when the first and the last exam scores are compared. This merely reflects that knowledge is effectively acquired over the course of learning using both teaching approaches. What is fundamentally different between the groups is that students that never or poorly attend classes performed worse when non-traditional methods are used because they do not improve exam scores longitudinally, in contrast with the control group.

Previous studies have demonstrated the impact of attendance on academic performance for traditional teaching [14-16]. In lecture-based physiology teaching, regular attendance was found to be helpful but not decisive [17]. Traditional methods for physiology teaching mostly include academic lectures, tutorials and laboratory practices. This teaching modality is easily followed by personal work as most available text books deal adequately with the material discussed in lectures. Therefore, students could easily compensate their absenteeism. In contrast, using new methodologies in Physiology teaching include several active-learning techniques and an integrated approach to gain a comprehensive understanding of all the physiological systems. Our data suggest that teacher supervision appears to be instrumental in order to achieve good academic performance in non-traditional teaching.

## Conclusions

We suggest that class attendance is critical when non-traditional active-learning methods are in use in Physiology teaching.

## Competing interests

The authors declare that they have no competing interests.

## Authors' contributions

BG, IB and CG conceived the study. BG and CG compiled the data. IB performed the statistical analysis. BG prepared and reviewed the manuscript. All authors read and approved the final manuscript
